# Atypical presentation of acute post-infectious glomerulonephritis in patients with sickle cell disease: report of two cases

**DOI:** 10.1186/s12882-020-01715-x

**Published:** 2020-02-24

**Authors:** Precil Diego Miranda de Menezes Neves, Bernardo Vergara Reichert, Ramaiane Aparecida Bridi, Luis Yu, Cristiane Bitencourt Dias, Rafaela Brito Bezerra Pinheiro, Leonardo de Abreu Testagrossa, Lívia Barreira Cavalcante, Denise Maria Avancini Costa Malheiros, Lectícia Barbosa Jorge, Viktoria Woronik

**Affiliations:** 1 Nephrology Division, University of São Paulo School of Medicine, Av. Dr. Enéas de Carvalho Aguiar, 255, 7° andar, Cerqueira Cesar, São Paulo, Brazil; 2 Pathology Division, University of São Paulo School of Medicine, São Paulo, Brazil

**Keywords:** Acute post-infectious glomerulonephritis, Nephrotic syndrome, Sickle cell anaemia

## Abstract

**Background:**

Sickle cell disease (SCD) is a highly prevalent genetic disease worldwide. In the natural evolution of SCD, glomerular lesions can develop, presenting histopathological patterns of segmental or focal membranoproliferative glomerulosclerosis, with or without thrombotic microangiopathy. We report two cases of acute post-infectious glomerulonephritis (APIGN), with atypical presentations, in patients with SCD.

**Case presentation:**

Case 1: An 18-year-old female with SCD presented with a 21-day history of progressive oedema, accompanied by dyspnoea, productive cough, fever, and chest pain. Blood tests showed the following: haemoglobin 6.1 g/dl; leucocytes 18,820 cells/mm^3^; and creatinine 0.49 mg/dl. A urine sample evidenced leucocyturia and haematuria. The 24-h proteinuria was 8.99 g, serum albumin level was 1.2 g/dl, low serum C3 levels and high levels of anti-streptolysin O. Renal biopsy was consistent with APIGN. The patient was treated with diuretic and anti-proteinuric agents, subsequently evolving to reversal of the renal alterations. Case 2: A 12-year-old male with SCD presented with a 20-day history of a non-productive cough and progressive oedema, together with hypertension. The serum creatinine concentration was 0.48 mg/dl. A urine sample evidenced leukocyturia and haematuria. The 24-h proteinuria was 12.5 g, and the serum albumin level was 2.6 g/dl. The levels of C3 and C4 were normal. Renal biopsy revealed APIGN. The patient was treated with diuretic and anti-proteinuric agents, subsequently evolving reversal of the renal alterations.

**Conclusions:**

The presentation of the two cases reported here are not typical of SCD-related kidney injury. Analysis of the renal biopsy specimens elucidated the diagnosis, affecting the prognosis, because that of APIGN is highly favourable, unlike that of nephrotic syndrome associated with SCD glomerulopathy.

## Background

Sickle cell disease (SCD) is one of the most prevalent monogenic diseases worldwide. The natural course of the disease can include the development of renal lesions, leading to haematuria, tubular disorders and possible progression to chronic kidney disease (CKD) due to intrinsic mechanisms of the disease. Proteinuria may be present and can reach the nephrotic range [1].

Renal impairment is a marker of poor prognosis in patients with SCD, due to the chance of progression to end-stage renal disease, which develops in up to 4.2% of cases [2]. Among patients with renal impairment secondary to SCD, there is also a higher incidence of other disease-related complications such as ischaemic stroke, gallstones, aseptic necrosis of the femoral head, and acute chest syndrome [3]. Nevertheless, it should be borne in mind that the appearance of nephrotic or nephritic syndrome, with or without a loss of renal function, in a patient with SCD is not always directly related to the underlying disease itself. Previous studies have shown that patients with SCD can develop glomerular diseases [4–8]. The fact that glomerular diseases can have atypical presentations in such patients makes renal biopsy indispensable for diagnostic clarification.

Renal abnormalities in pediatric SCD patients may be detected even in the first decade of life. The primary observed changes are increased renal blood flow, which leads to glomerular hyperfiltration and hypertrophy, conducing along the time to loss of glomerular basement membrane selectivity and, consequently, albuminuria and proteinuria [9, 10]. It is estimated that about 16–27% of children have albuminuria, and levels of albuminuria> 500 mg/g and nephrotic syndrome are associated with faster progression to end-stage renal disease in adulthood. In adolescence, glomerular filtration rate < 90 ml/min/1.73 m2 is already observed in 10% of SCD patients [9–11].

Acute Post-Infectious Glomerulonephritis (APIGN) is characterized by the development of an acute glomerulopathy after exposure to microorganisms. In its classic form, APIGN is associated with skin and/or upper airway infection by group A β-hemolytic streptococci and it mainly affects children aged to 4–14 years. Its incidence in developed countries is becoming less frequent in the scenario of better health and hygiene conditions. The disease is clinically manifested as the classic prototype nephritic syndrome, with haematuria, hypertension, lower limb edema and sub-nephrotic proteinuria [12, 13]. Cases with atypical presentation such as nephrotic syndrome or rapidly progressive glomerulonephritis are described, usually affecting patients with other underlying diseases (alcoholism, diabetes, elderly) but they correspond to the minority of cases [14, 15].

In the present article, we report the cases of two patients with SCD and nephrotic syndrome. In both cases, the analysis of the renal biopsy specimen showed Acute Post-Infectious Glomerulonephritis (APIGN) and the clinical presentation of the APIGN was atypical.

## Case presentation

### Case 1

An 18-year-old Black female who had been diagnosed with SCD in childhood, subsequently suffering frequent pain crises and undergoing repeated transfusions, presented to the emergency room with a 21-day history of progressive lower limb oedema accompanied by dyspnoea and orthopnoea. In addition, she reported a 3-day history of severe chest pain, together with a productive cough and fever as high as 38 °C. She reported no reduction in urine volume and no recent history of skin infection or tonsillitis. On physical examination, she was found to be very pallid and normotensive (blood pressure, 130/80 mmHg), with a heart rate of 104 bpm, a respiratory rate of 28 breaths/min, and oxygen saturation of 94% on room air, as well as being found to have developed anasarca. One month prior, she had undergone outpatient laboratory tests, which had shown that her renal function was normal—with a serum creatinine concentration of 0.75 mg/dl and an estimated glomerular filtration rate (eGFR), as determined by the Chronic Kidney Disease–Epidemiology Collaboration (CKD–EPI) equation, of 134.9 ml/min/1.73 m^2^—urine analysis showing no haematuria or proteinuria.

Blood tests performed at admission to the emergency room revealed the following: haemoglobin, 6.1 g/dl; haematocrit, 18.8%; haptoglobin, < 10 mg/dl; reticulocyte fraction, 6.11%; no schistocytes; lactate dehydrogenase, 433 U/L; indirect bilirubin, 0.36 mg/dl; leucocyte count, 18,820 cells/mm^3^; neutrophil count, 13,630 cells/mm^3^; lymphocyte count, 2790 cells/mm^3^; platelet count, 622;000 cells/mm^3^; and urea, 29 mg/dl. The patient showed signs of hyperfiltration, including a serum creatinine concentration of 0.49 mg/dl and a CKD–EPI-determined eGFR of 148.5 ml/min/1.73 m^2^, although no hydroelectrolytic or acid-base disorders were identified. A urine sample was found to contain 100 leucocytes/field and 70 erythrocytes/field. In addition, 24-h proteinuria was 8.99 g. Further analysis of the blood samples showed the following: total proteins, 3.9 g/dl; albumin, 1.2 g/dl; globulins, 2.7 g/dl; total cholesterol, 210 mg/dl, low-density lipoprotein, 114 mg/dl; high-density lipoprotein, 37 mg/dl; triglycerides, 205 mg/dl; C4, 26.1 mg/dl (normal range, 10–38 mg/dl); C3, 62 mg/dl (normal range, 73–149 mg/dl); C-reactive protein, 24.9 mg/dl (normal range, < 5 mg/dl). The serology was negative for HIV, hepatitis B, and hepatitis C. The patient also tested negative for antinuclear factor, anti-DNA, rheumatoid factor, and antineutrophil cytoplasmic antibodies. Serum protein electrophoresis showed hypoalbuminaemia and an anti-streptolysin O level of 816 IU/ml (normal range, < 200 IU/ml). Blood and urine cultures were negative. A chest X-ray showed mild congestion and infiltrate in the right lung base. Ultrasound of the kidneys and urinary tract showed that the kidneys were normal in size and appearance.

Given the clinical presentation, the diagnostic hypothesis was pneumonia and anasarca due to nephritic-nephrotic syndrome. The patient was started on ceftriaxone (1 g, twice daily). Computed tomography angiography of the chest ruled out pulmonary thromboembolism and acute chest syndrome, thus indirectly confirming the diagnosis of pneumonia.

**Fig. 1:**
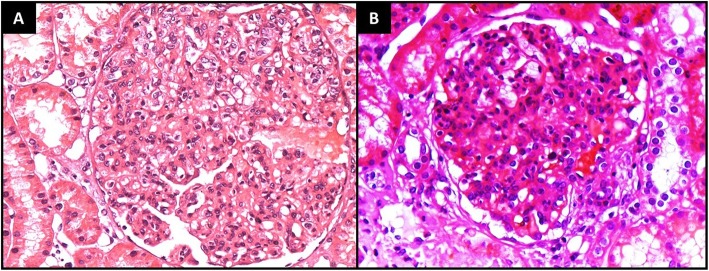
**a** High-power light microscopy view of a large glomerulus with global capillary occlusion caused by endocapillary proliferation, together with neutrophil infiltration. (Haematoxylin and eosin; magnification, × 400).**b** Photomicrograph showing global endocapillary and mesangial hypercellularity in a glomerulus with neutrophils. (Haematoxylin and eosin; magnification, × 400)

After the infection had been controlled, the patient underwent renal biopsy. Light microscopy revealed 7 glomeruli with global endocapillary proliferation; neutrophil and macrophage infiltrate (Fig. 1a); synechiae in 10% of the glomeruli; occasional splitting of the glomerular basement membrane; and a preserved Bowman’s capsule. The tubules were dilated, with foci of epithelial regeneration and neutrophils within their lumina. There was focal oedema in the interstitium. The interlobular arteries had a normal aspect, with intimal proliferation in the arterioles. Immunofluorescence performed on frozen sections showed intensity of deposition of C3 (2+/3+), IgG (1+/3+), IgA (1+/3+) and lambda light chains (1+/3+), with a granular pattern, distributed diffusely throughout the capillary loop. Collectively, the histological findings were consistent with a diagnosis of APIGN accompanied by microangiopathic changes related to the underlying disease.

**Fig. 2:**
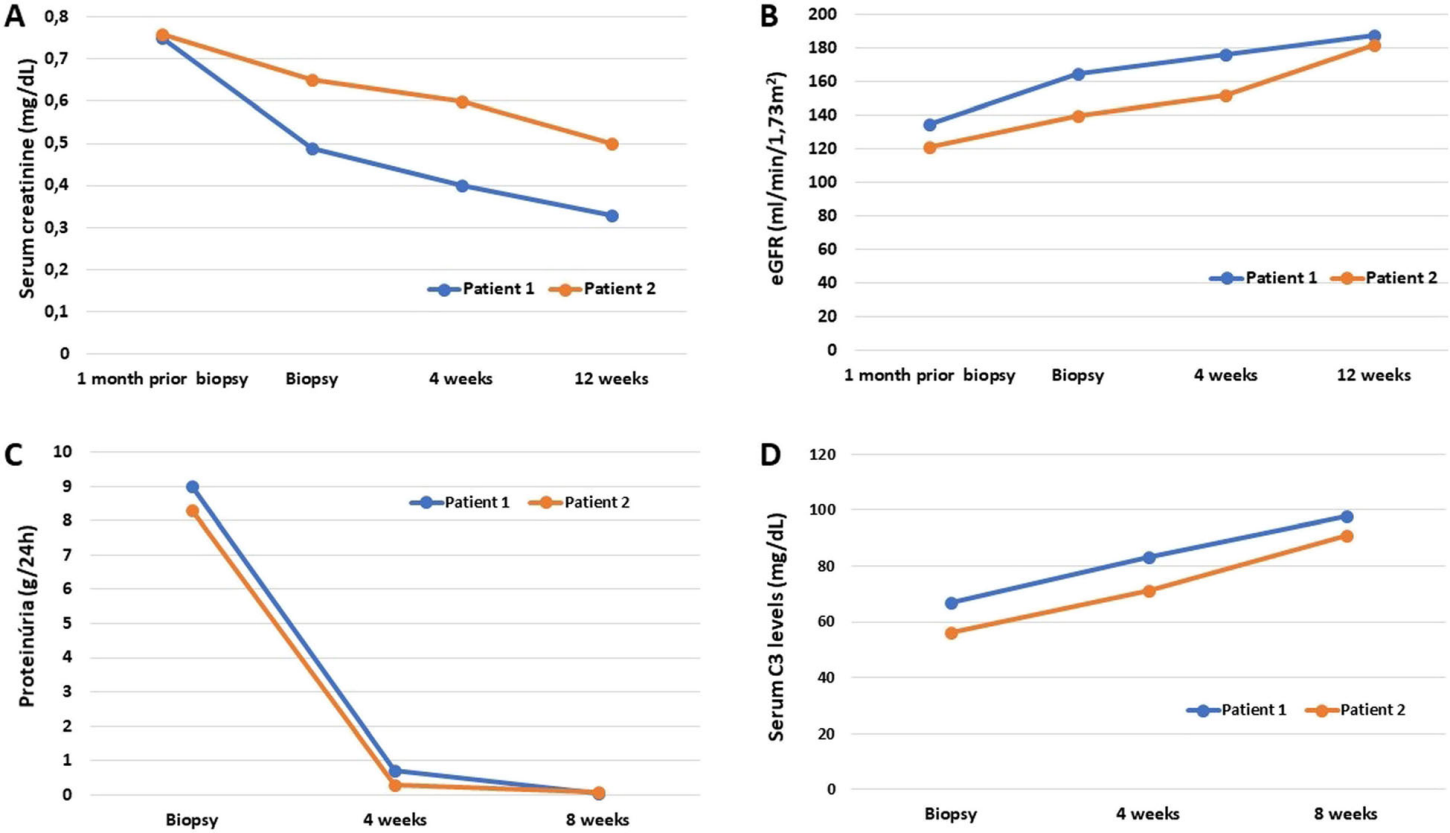
Evolution of laboratory tests for 12 weeks **a** Serum creatinine; **b** eGFR (Estimated glomerular filtration rate). **c** 24-h proteinuria and **d** Serum C3 levels (normal range: 73-149 mg/dL)

Treatment with a diuretic and an anti-proteinuric agent (angiotensin-converting enzyme inhibitor) was instituted, and the patient evolved to clinical improvement (normalisation of blood pressure, as well as resolution of the proteinuria and haematuria). However, the signs of hyperfiltration persisted even after resolution of the proteinuria. In the final evaluation, the serum creatinine concentration was 0.33 mg/dl and the CKD–EPI-determined eGFR was 187.8 ml/min/1.73 m^2^. Evolution of laboratorial tests are available in Fig. 2.

### Case 2

A 12-year-old White male, who had also been diagnosed with SCD in childhood, sought treatment for a 20-day history of non-productive cough and lower limb oedema, which had evolved to anasarca, together with a new onset of hypertension. He had previously experienced one episode of pain and had undergone one transfusion. He reported no reduction in urine volume and no recent history of skin infection or tonsillitis. On physical examination, he was found to be pallid (+ 3/+ 4) and hypertensive (blood pressure, 160/90 mmHg), with a heart rate of 112 bpm, a respiratory rate of 36 breaths/min, and oxygen saturation of 92% on room air.

Blood tests performed at admission to the emergency room revealed the following: haemoglobin, 5.9 g/dl; haematocrit, 17.8%; haptoglobin, < 10 mg/dl; negative result on the Coombs direct antibody test; reticulocyte fraction, 4.26%; no schistocytes; lactate dehydrogenase, 970 U/L; indirect bilirubin, 0.65 mg/dl; leucocyte count, 17,330 cells/mm^3^; neutrophil count, 13,100 cells/mm^3^; lymphocyte count, 1700 cells/mm^3^; platelet count, 622;000 cells/mm^3^; and urea, 45 mg/dl. He also showed signs of hyperfiltration, with a serum creatinine concentration of 0.65 mg/dl, and an eGFR, as determined with the Schwartz formula, of 140.0 ml/min/1.73 m^2^, although no hydroelectrolytic or acid-base disorders were identified. An urine sample obtained at submission was found to contain 20 leucocytes/field and > 100 erythrocytes/field; 24-h proteinuria was 12.5 g. Metabolic analysis of the blood samples showed the following: total proteins, 6.1 g/dl; albumin, 2.6 g/dl; globulins, 3.1 g/dl; total cholesterol, 211 mg/dl, low-density lipoprotein, 118 mg/dl; high-density lipoprotein, 66 mg/dl; triglycerides, 133 mg/dl; C4, 25 mg/dl (normal range, 10–38 mg/dl); C3, 106 mg/dl (normal range, 73–149 mg/dl); C-reactive protein, 2.1 mg/dl (normal range, < 5 mg/dl). The serology was negative for HIV, hepatitis B, and hepatitis C. Tests for antinuclear factor, anti-DNA, rheumatoid factor, and antineutrophil cytoplasmic antibodies were also negative. Serum protein electrophoresis showed hypoalbuminaemia. Blood and urine cultures were negative. A chest X-ray showed cardiomegaly and signs of mild pulmonary congestion. Ultrasound of the kidneys and urinary tract showed that the kidneys were normal in size and appearance.

The patient underwent renal biopsy. Light microscopy showed 43 glomeruli with diffuse endocapillary hypercellularity (numerous lymphocytes and neutrophils). Synechiae were observed in 10% of the glomeruli in Bowman’s capsule. There was also occasional splitting of the glomerular basement membrane, as well as overall expansion of the mesangial matrix (Fig. 1b). There were no changes in the tubules, interstitium or arterioles. Immunofluorescence showed deposition of C3 (2+/3+) and lambda light chains (1+/3+), with a granular pattern, distributed throughout the capillary loop—the so-called “starry sky” pattern—consistent with a diagnosis of APIGN.

Treatment with a diuretic and an anti-proteinuric agent (angiotensin-converting enzyme inhibitor) was instituted, and the patient evolved to clinical improvement, including normalisation of blood pressure and a significant reduction in the level of proteinuria (24-h proteinuria, 1.34 g) after 10 days. Renal function was preserved. The patient left outpatient treatment after 18 months. In the final evaluation, he still presented no proteinuria or haematuria, although the signs of hyperfiltration persisted, his serum creatinine concentration being 0.5 mg/dl and his Schwartz-derived eGFR being 182 ml/min/1.73 m^2^. Evolution of laboratorial tests are available in Fig. 2.

## Discussion and conclusions

In patients with SCD, kidney injury can involve various pathophysiological substrates, the most common clinical manifestations being haematuria and proteinuria, with progression to CKD. Nath et al. [16] proposed the phenomenon of the “perfusion paradox” where the relative hypoxia, acidosis and hyperosmolarity found in the microcirculation of the renal medullary region would favour red blood cell sickling, producing medullary infarctions resulting in reduced blood supply, as well as the release of prostaglandins and kallikreins, producing glomerular hyperfiltration. The hyperfiltration in turn leads to glomerular hypertrophy and glomerular hypertension, with subsequent proteinuria and evolution to glomerulosclerosis [17–20], and nephrotic syndrome has been reported to occur in 20–40% of patients with SCD [17]. The main findings in renal biopsies of patients with SCD are segmental or focal glomerulosclerosis, a membranoproliferative pattern, non-immunological splitting or thickening of the glomerular basement membrane, thrombotic microangiopathy, collapse of capillary loops, sickle cells in the lumina of the medullary capillaries, and haemosiderin deposits [21]. Despite this intrinsic relationship between SCD and the development of nephropathies, other glomerulopathies cannot be immediately ruled out in this population, especially when the clinical presentation is not completely consistent with a diagnosis of APIGN.

Acute Post-Infectious Glomerulonephritis is a disease that classically affects children among between 4 and 14 years and it occurs most commonly after skin or upper airway infection by group A β-hemolytic streptococci. In its usual course, APIGN presents with lower limb edema, hypertension, haematuria, non-nephrotic proteinuria, however some atypical cases that may progress to rapidly progressive glomerulonephritis or present with nephrotic proteinuria and/or nephrotic syndrome. There is usually activation of the complement alternative pathway, evidenced by the consumption of C3 [12, 13]. APIGN treatment is supportive, with low salt diet, antihypertensive therapy, diuretics, and antibiotic treatment to eradicate the nephritogenic strain. Immunosuppression is only indicated in cases of rapidly progressive course. In both cases we opted for the initiation of ACE inhibitor since the patients did not show progressive worsening of renal function during the event, hydroelectrolytic disorders or significant reduction in urinary volume. In addition, there are some studies [22, 23] showing that the use of ACE inhibitor promoted better blood pressure control compared to other classes of anti- hypertensive. Regarding the use of steroids for treatment, we chose to wait for the biopsy result to follow guided therapy, since both patients were treating infection beyond the fact that they did not present significant worsening of renal function. The disease is usually self-limiting, with clinical resolution and normalization of laboratory tests in about 6–8 weeks. In children with typical clinical features, renal biopsy may be dispensed, however, atypical clinical presentation or non-resolution of clinical and laboratory abnormalities within 8 weeks are indications of renal biopsy [13, 15]. In the presented cases, both patients already had evidence of renal hyperfiltration before acute APIGN (Patient 1: 134.9 ml/min/1,73 m2 and Patient 2: 121 ml/min/1,73 m2). In this context of a pre-existing structural change caused by sickle cell anemia, we believe that inflammatory changes would act on glomerular permeability and exacerbate this hyperfiltration and trigger a significant increase overlapping sub-nephrotic proteinuria already commonly observed in patients with APIGN.

In recent years, the profile of APIGN has been changing, and the incidence of the disease in patients with other comorbidities such as diabetes, alcoholism and elderly patients has increased. In such situations, several other types of microorganisms may be associated with the disease, there may be atypical clinical manifestations (such as nephrotic proteinuria and/or nephrotic syndrome), different histological findings such as predominance of IgA to immunofluorescence, as well as a higher chance of evolution to chronic kidney disease [24–26].

The presentations of the two cases reported here, in patients with few SCD-related extra-renal complications, no previous proteinuria and developing acute nephritic-nephrotic syndrome, are not typical of the kidney injury associated with SCD. In these cases, renal biopsy was important for diagnostic clarification, directly affecting the prognosis, because that of APIGN is highly favourable, unlike that of nephrotic syndrome associated with SCD glomerulopathy. Occasional splitting and wrinkling of the glomerular basement membrane are common in SCD, reflecting chronic ischaemia, and can often create confusion for a pathologist evaluating a biopsy specimen obtained from a patient with SCD [21, 27].

There have been few reports of APIGN in patients with SCD [4–7]. Assar et al. [4] presented a case, similar to one reported here, in which the patient had pure nephrotic syndrome, without complement consumption, emphasising the difficulty in differentiating these cases with SCD-related nephrotic-range proteinuria. Roy et al. [5] reported three cases, which presented clinically as pure, nephritic and mixed nephrotic syndrome, respectively. Susmano et al. [6], reported two cases of apparently nephritic syndrome, and Pashankar et al. [7] reported a similar case, in which the patient developed reversible posterior leucoencephalopathy secondary to hypertension. Unlike our two patients, those in question were children. However, as in our cases, the evolution was favourable, with complete reversal of the clinical and biochemical changes associated with glomerulopathy, in all of those cases. Previous streptococcal infection was the precipitating factor in most of those cases.

In conclusion, primary glomerulopathies can be difficult to diagnose in patients with SCD, because the findings can be similar to those of SCD-related nephropathy and because the manifestations can be atypical in such patients. Renal biopsy is recognised as an indispensable tool for making an accurate diagnosis in these situations.

## Data Availability

All meaningful data generated or analyzed in this study are included in the manuscript.

## Abbreviations

APIGN: Acute Post-Infectious glomerulonephritis
CKD–EPI: Chronic Kidney Disease–Epidemiology Collaboration
eGFR: Estimated glomerular filtration rate
SCD: Sickle cell disease
